# Tuning Mechanical Properties of Pseudopeptide Supramolecular Hydrogels by Graphene Doping

**DOI:** 10.3390/molecules24234345

**Published:** 2019-11-28

**Authors:** Demetra Giuri, Marianna Barbalinardo, Nicola Zanna, Paolo Paci, Marco Montalti, Massimiliano Cavallini, Francesco Valle, Matteo Calvaresi, Claudia Tomasini

**Affiliations:** 1 Dipartimento di Chimica “Giacomo Ciamician”, Università di Bologna, Via Selmi, 240126 Bologna, Italy; demetra.giuri2@unibo.it (D.G.); nicola.zanna@studio.unibo.it (N.Z.); paolo.paci@studio.unibo.it (P.P.); marco.montalti2@unibo.it (M.M.); 2 Istituto per lo Studio dei Materiali Nanostrutturati, Consiglio Nazionale delle Ricerche, (ISMN-CNR), Via P. Gobetti 101, 40129 Bologna, Italy; m.barbalinardo@bo.ismn.cnr.it (M.B.); massimiliano.cavallini@cnr.it (M.C.); francesco.valle@cnr.it (F.V.)

**Keywords:** hydrogels, graphene, rheology, self-healing, thixotropy

## Abstract

Supramolecular hydrogels, obtained from small organic molecules, may be advantageous over polymeric ones for several applications, because these materials have some peculiar properties that differentiate them from the traditional polymeric hydrogels, such as elasticity, thixotropy, self-healing propensity, and biocompatibility. We report here the preparation of strong supramolecular pseudopeptide-based hydrogels that owe their strength to the introduction of graphene in the gelling mixture. These materials proved to be strong, stable, thermoreversible and elastic. The concentration of the gelator, the degree of graphene doping, and the nature of the trigger are crucial to get hydrogels with the desired properties, where a high storage modulus coexists with a good thixotropic behavior. Finally, NIH-3T3 cells were used to evaluate the cell response to the presence of the most promising hydrogels. The hydrogels biocompatibility remains good, if a small degree of graphene doping is introduced.

## 1. Introduction

Hydrogels have been extensively explored as promising biomaterials for biosensors, tissue engineering, and drug delivery systems [1,2,3,4], because of their great biocompatibility, environmental responsiveness, and tunable mechanical and bioactive properties mimicking natural extracellular matrices [5,6,7,8]. Low-molecular-weight supramolecular hydrogels (LMWSH) arise from the self-assembly of small molecules into long, anisotropic structures. At a sufficiently high concentration, these structures entangle leading to a supramolecular network that is able to immobilize the solvent. Low-molecular-weight supramolecular hydrogels [9,10,11] strongly differ from polymeric gels and may be advantageous over polymeric ones for several applications, because these materials have some peculiar properties that differentiate them from the traditional polymeric hydrogels, such as elasticity, thixotropy, self-healing propensity, and thermoreversibility.

Peptide-based hydrogels [12,13,14,15,16], are particularly appealing for biomedical applications, because they provide various advantages including high biocompatibility and good biodegradability. However, many supramolecular hydrogels have low mechanical stability and high erosion rates, and therefore are unstable in vivo [17]. This greatly hinders their application. Hydrogels based on pseudopeptides give the peptide hydrogel additional intrinsic proteolytic stability.

A strategy that has emerged for improving the functionality of supramolecular hydrogels is the incorporation of nanofillers [18,19,20,21,22,23], applied to exploit the specific properties of the fillers to either modify the performance of the hydrogel or add functionality (i.e., hydrogel reinforcement and self-healing) [24,25,26,27], as long as they do not hamper molecular self-assembly. Nanocarbons are ideal nanofillers due to their peculiar chemical–physical properties, which have demonstrated great potential in nanomedicine and nanotechnology [28,29,30,31]. The electrical conductivity, mechanical strength, and high surface area make graphene one of the most interesting fillers for tissue engineering and regenerative medicine [32,33,34,35]. The generally poor mechanical stability of hydrogels limits their use as functional materials for many biomedical applications. Graphene is the strongest material known. It is more than 200 times stronger than steel. The presence of graphene, even at very low loadings, can provide significant reinforcement to the final material, so we investigated the effect of graphene doping inside our hydrogels.

Recently, incorporation of graphene-based nanofillers in hydrogels has been used to tailor their properties [36,37,38,39,40,41]. A crucial step for the inclusion of graphene in hydrogels is the achievement of a stable dispersion. Various polymers/surfactants/biomolecules were used to facilitate the dispersion of carbon nanomaterials [42,43,44,45] in solution, but these dispersants usually alter the gelation process. The best solution is the dispersion of graphene using directly the supramolecular gel medium. Aromatic moieties contained in the gelator molecules can interact with the graphene delocalized π surface of the by π–π stacking interactions, which are crucial for the dispersion of graphene in the supramolecular matrix and for the incorporation of the graphene sheets into the gel system, generating a hybrid system. Moreover, the supramolecular gel materials provide fibrillar network structures that may accommodate the dispersed graphene sheets [46].

We recently reported several pseudopeptides that freeze water in low concentration and form hydrogels that possess high mechanical strength and transparency [47,48,49]. All these molecules showed a good propensity to self-assemble in water, due to the presence of the d-Oxd or d-pGlu moiety [Oxd = (4*R*,5*S*)-4-methyl-5-carboxyl-oxazolidin-2-one, pGlu = pyroglutamic acid] in their skeleton [50,51] that imposes a constraint to the chain, together with the presence of aromatic rings contained in aromatic amino acids, such as Phe or Tyr (Phe: phenylalanine; Tyr: tyrosine). These pseudopeptides easily form fibers [52,53] that self-assemble to yield hydrogels of different strength, pH, and transparency [49,54,55,56,57]. Several techniques may promote the gelation process of these molecules: salt addition [56,58,59], pH variation [60,61], enzymatic cleavage [62,63], dissolution in solvent mixtures [64], and ultrasound sonication [65,66]. 

**Figure 1:**
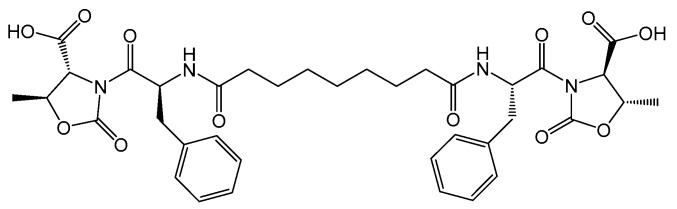
Chemical structure of the gelator **A** analyzed in this study.

The already reported bolamphiphilic pseudopeptide HO-d-Oxd-l-Phe-CO(CH_2_)_7_CO-l-Phe-d-Oxd-OH **A** possesses two l-Phe-d-Oxd (Phe = phenylalanine) dipeptide units coupled with an azelaic acid unit (Figure 1) [66]. Molecule **A** is an efficient gelator and contains the aromatic moieties of l-Phe that can interact with graphene and help to disperse it.

In this work, the pseudopeptide **A** was used both as gelator and graphene dispersant to prepare a small library of graphene doped supramolecular hydrogels. Two different triggers were used for the gelation (pH variation and arginine addition), and the hydrogels mechanical properties were tested and compared. The hydrogels biocompatibility was also tested.

## 2. Results and Discussion

We prepared 16 hydrogels using solutions of gelator **A** (in 1% or in 2% *w*/*w* concentration) and an increasing amount of graphene in MilliQ water (see Table 1 for details), adding either glucono-δ-lactone (GdL) (2.2 equivalents) or arginine (Arg) (1 equivalent) to promote the hydrogel formation.

Although the use of GdL has been extensively reported to promote gel formation [60,67,68], the choice of arginine as trigger was suggested by our previous work [49], where we compared the properties of several amino acids, and arginine proved to be the reagent of choice among them. The 16 mixtures were stirred for 5–10 min, then left stand in the test tube overnight until gel formation (for the photographs of hydrogels **1**–**16**, see Figure S1). The hydrogels are thermally stable, self-supporting and thermoreversible on heating, with T_gel_ increasing for hydrogels doped with graphene increasing amounts (Table 1). Hydrogels **1**–**8** have pH values ranging between 3.5 and 4 due to the use of GdL as trigger, whereas hydrogels **9**–**16** reach a pH ranging between 7 and 8 due to the addition of arginine.

**Figure 2:**
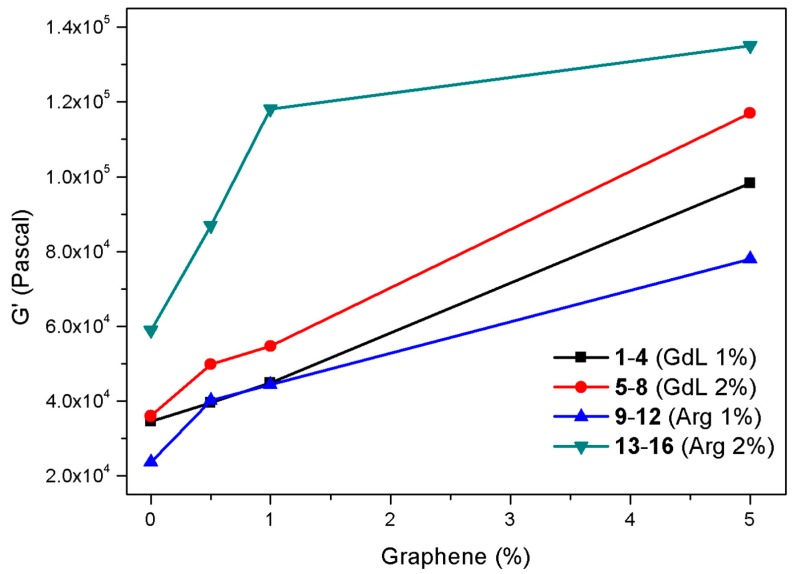
Diagram of the G’ values obtained from frequency sweep analyses of hydrogels **1**–**16** (ω = 0.2) as a function of the trigger, gelator concentration, and graphene concentration.

To analyze the viscoelastic behavior of hydrogels **1**–**16**, rheological analyses have been performed to evaluate them in terms of storage (G’) and loss moduli (G″). All the analyzed hydrogels are characterized by a “solid-like” behavior, i.e., the storage modulus is approximately an order of magnitude higher than the loss component (see Table S1 for details). If we compare the properties of hydrogels obtained at both 1% and 2% *w*/*w* concentration, we can gather that G’ always increases as the graphene concentration increases (Figure 2); this effect is more pronounced for arginine containing hydrogels **13**–**16**.

Considering the mechanical properties and the final pH of the 16 samples, we can gather that the most promising hydrogels for applications as biocompatible materials are hydrogels **13**–**16** that couple an exceptional mechanical strength with a neutral pH (they all have a final pH ranging between 7 and 8) that is compatible with cellular environment. Thus, we further analyzed all the hydrogels, with a particular interest in the properties of hydrogels **13**–**16**.

Self-healing properties [49,69,70,71,72,73] may be defined as the ability to autonomously reconstruct the bonding interactions after damage by a step strain experiment, whereas thixotropic behavior [72,74,75,76] means that the gel becomes liquid if a shear stress is applied, then quickly recovers the solid form on resting. To test the self-healing and thixotropic properties of samples **1**–**16**, multiple cycles composed of three steps were applied to the gels. During the first step, the sample was subjected to a strain value within the LVE region and was characterized by G’ values greater than G’’. When the applied strain was increased above the crossover point, the sample behavior switched from gel-like to sol-like, with G’’ values greater than G’. Finally, the sample was leaved at fixed strain within the LVE range to check the recovery of the gel-like behavior.

**Figure 3:**
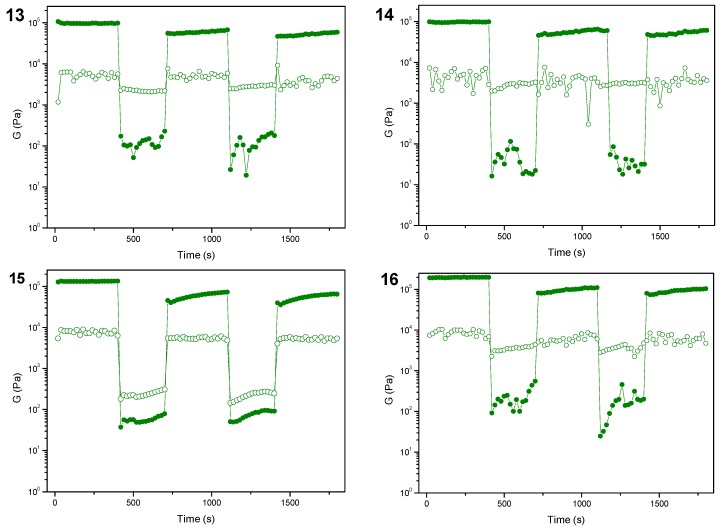
Values of storage moduli (solid circles) and loss moduli (empty circles) during a step strain experiment performed on hydrogels **13**–**16**.

Hydrogels **13**–**16** are characterized by a great capability to recover the gel-like behavior and confirm at the molecular level their thixotropic properties, as they all fully recover the “solid-like” behavior when the strain level goes back within the LVE region (Figure 3). The results observed for hydrogels **1**–**8** (Figure S2) show that they are all very fragile, as they are characterized by a modest capability to recover to gel-like behavior after the application of strain well above its LVE region. In contrast, hydrogels **9**–**12** provided very good results, proving to be more elastic (Figure S3).

**Figure 4:**
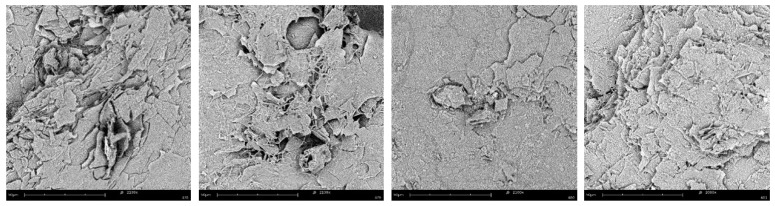
From left to right: SEM images of aerogel obtained by freeze drying hydrogels samples **13**–**16**.

Much information on the nature of the hydrogels was obtained by SEM analysis of the corresponding aerogels obtained by freeze-drying these samples. In Figure 4 the images of aerogels **13**–**16** are reported, whereas all the aerogels images are shown in Figure S4.

In any case, the comparison between aerogels containing graphene and the corresponding aerogels without graphene do not suggest significant differences due to graphene, as aerogels **13**–**16** (Figure 4) and **1**–**8** (Figure S4) show a morphology characterized by the formation of locally oriented long strips that cross on the large scale, thus forming a network, whereas aerogels **9**–**12** are characterized by dense fibrous networks (Figure S4). 

**Figure 5:**
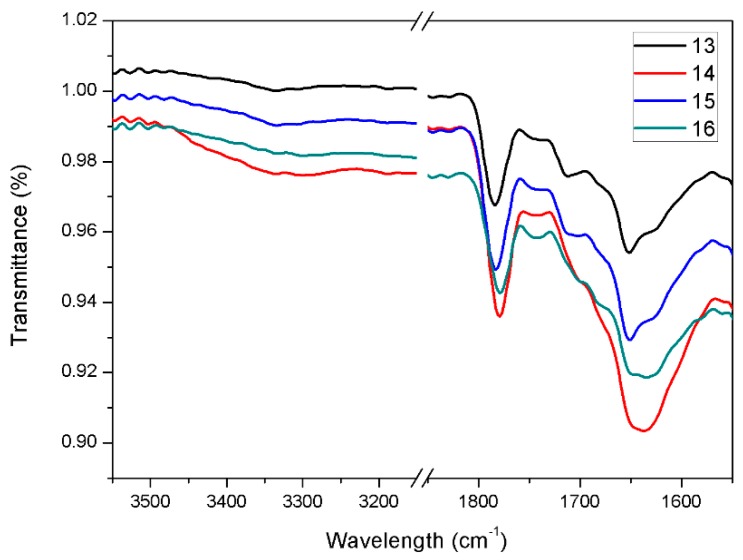
Selected regions of attenuated total reflection infrared spectroscopy (ATR-IR) spectra of aerogels **13**–**16**.

This outcome is further confirmed by the analysis of the aerogels ATR-IR spectra (Figure 5 and Figure S5), which provides information on the supramolecular interactions involved in the formation of the hydrogels. The spectra of hydrogels prepared with the same gelator concentration and trigger are compared, varying the graphene concentration. In all the graphs, very similar spectra are always obtained. 

**Figure 6:**
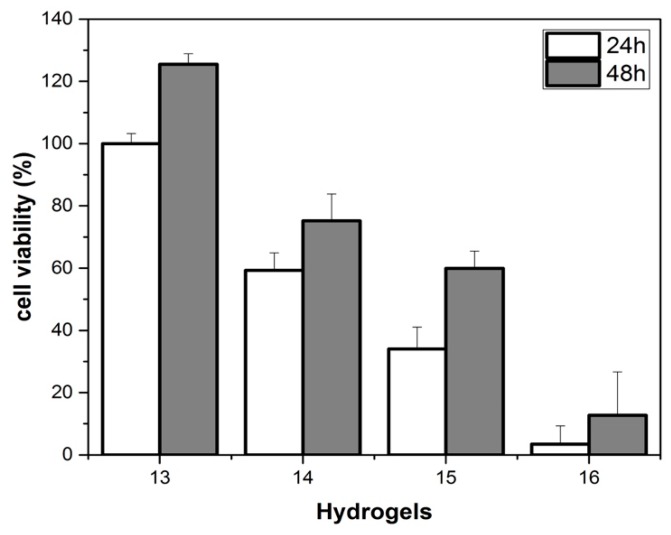
Cell viability of NIH-3T3 on hydrogels **13**–**16** in cellular medium. Data represent the mean ± standard deviation.

NIH-3T3 cells were used to evaluate the cell response to the presence of hydrogels **13**–**16**. We manufactured the hydrogels using medium cellular instead of MilliQ water, to assess the hydrogels biocompatibility. Use of cellular medium slowed down the gelation time from 16 h to 2 days. Figure 6 illustrates cell proliferation on hydrogels **13**–**16** in cellular medium after a culture period of 24 h and 48 h.

Cell adhesion is strongly influenced by graphene doping. Hydrogel **14** shows good biocompatibility, whereas at higher graphene concentrations (hydrogels **15** and **16**), a significant toxic effect on fibroblasts is observed. However, after 48 h the cells continue to proliferate both in hydrogel **13** (without graphene) and in hydrogel **14**–**16**, insensitive to the graphene doping concentration. This mean that the scaffold support cell growth. Supramolecular hydrogel, doped with graphene, may represent a promising building block for the development of smart materials for biomolecules and tissue engineering purposes. The degradation of the gel does not modify the graphene toxicity. We measured the cell viability after the destruction of the matrix, and the results, in Figure S8, showed that cell viability is quite similar to the one obtained by the graphene embedded in the gel.

## 3. Materials and Methods

### 3.1. Materials

All chemicals and solvents were purchased from Sigma-Aldrich (St. Louis, MO, USA), VWR (Radnor, PA, USA), or Iris Biotech GMBH (Marktredwitz, Germany) and used as received. Acetonitrile was distilled under inert atmosphere before use. MilliQ water (Millipore, resistivity = 18.2 mΩ.cm) was used throughout. Solvent were dried by distillation before use. All reactions were carried out in dried glassware.

### 3.2. Synthesis of HO-d-Oxd-l-Phe-CO(CH_2_)_7_CO-L-Phe-D-Oxd-OH (Gelator **A**)

The compound was synthesized from d-Thr, l-Phe, and azelaic acid following a multistep procedure in solution, reported in [49,66]

### 3.3. Preparation of the Graphene Dispersions

Three dispersions of MilliQ water (20 mL each) containing edge-functionalized graphene, (10 mg, 20 mg, and 100 mg, respectively) were prepared and vigorously stirred for ~20 min with a magnetic stirrer. The graphene sample, produced by 24 h ball-milling in the presence of CO_2_ according to [77], was kindly provided by the University of Fribourg. Characterization of the graphene sheets (elemental analysis, surface area, and size of the sheets) are reported in [77].

### 3.4. Hydrogel Preparation using the pH Variation Method

A portion of gelator **A** (5 mg for hydrogels **1**–**4**; 10 mg for hydrogels **5**–**8**) was placed in a test tube (diameter: 8 mm), then MilliQ water (0.5 mL) or a graphene solution (see Table 1 for details) was added, followed by a 1M aqueous NaOH (2.1 equivalent) and the mixture was stirred until sample dissolution. Finally, we added 2.2 equivalents of glucono-δ-lactone (GdL). After a rapid mixing to allow the GdL complete dissolution, the sample could stand quiescently until complete gel formation which usually occurred after 16 h. 

### 3.5. Hydrogel Preparation by Addition of Arginine

A portion of gelator **A** (5 mg for hydrogels **9**–**12**; 10 mg for hydrogels **13**–**16**) was placed in a test tube (diameter: 8 mm), then arginine (1 equivalent) was added. 0.5 mL of MilliQ water or a graphene solution (see Table 1 for details) was added to the test tube under stirring. After 1 min, the magnetic stirrer was removed and the tube could stand quiescently until complete gel formation which usually occurred after 16 h.

### 3.6. Conditions for T_gel_ Determination

T_gel_ was determined by heating some test tubes (diameter: 8 mm) containing the gel and a glass ball (diameter: 5 mm; weight: 165 mg) on the top of it. When the gel is formed, the ball is suspended atop. The T_gel_ is the temperatures in which the ball starts to penetrate inside the gel. Some hydrogel samples melt, producing a clear solution, whereas in other cases, the gelator shrinks and water is ejected, as syneresis occurs.

### 3.7. Aerogels Preparation

Samples of hydrogels **1**–**16** were freeze-dried using a BENCHTOP Freeze Dry System LABCONCO 7740030 (LABCONCO, Kansas City, KS, USA) with the following procedure; the hydrogel was prepared into an Eppendorf test tube at room temperature. After 16 h, the samples were deepened in liquid nitrogen for 10 min, then freeze-dried for 24 h in vacuo (0.2 mBar) at −50 °C.

### 3.8. Aerogels Characterization

#### 3.8.1. Morphological Analysis

Scanning electron micrographs of the samples were recorded using a Hitachi 6400 field emission gun scanning electron microscope operating at 15 kV (Hitachi, Chiyoda, Tokyo, Japan).

#### 3.8.2. Rheology

Rheology experiments were carried out on an Anton Paar Rheometer MCR 102 (Anton Paar, Graz, Austria) using a parallel plate configuration (25 mm diameter). Experiments were performed at constant temperature of 23 °C controlled by the integrated Peltier system and a Julabo AWC100 cooling system. Oscillatory amplitude sweep experiments (γ: 0.01–100%) were carried out to determine the linear viscoelastic (LVE) range at a fixed frequency of 1 rad s^−1^. Once the LVE of each hydrogel was established, frequency sweep tests were performed (ω: 0.1–100 rad s^−1^) at a constant strain within the LVE region of each sample (γ = 0.04%). Step strain experiments were performed on hydrogels to analyze the thixotropic behavior of the material. The sample was subjected to consecutive deformation and recovery steps. The recovery step was performed by keeping the sample at a constant strain γ = 0.04%, i.e., within the LVE region, for a period of 400 s. The deformation step was performed by applying to the gel a constant strain γ = 100%, i.e., above the LVE region of the sample for a period of 300 s. The cycles were performed 3 times at a fixed frequency ω = 10 rad s^−1^.

#### 3.8.3. IR

High quality infrared spectra (64 scans) were obtained with an ATR-FT-IR Bruker Alpha System spectrometer (64 scans) (Bruker, Billerica, MA, USA).

### 3.9. Cell Culture

Mouse embryonic fibroblast (NIH-3T3) cells were cultured under standard conditions in Dulbecco’s modified Eagle’s medium (DMEM) (4.5 g L^−1^ glucose), supplemented with 10% (*v*/*v*) Fetal Bovine Serum, 2 mM l-glutamine, 0.1 mM MEM Nonessential Amino Acids (NEAA), 100 U mL^−1^ penicillin, and 100 U mL^−1^ streptomycin in a humidified incubator set at 37 °C with 5% CO_2_. For cell culture, the samples were sterilized with ultraviolet (UV) radiation for 20 min before use. The gels were incubated for 30 min at 37 °C with 5% CO_2_ with complete medium. Cells were seeded on gel in 24-well plates at a density of 10^5^ cells per mL.

### 3.10. Cell Viability

Cell viability was determined by resazurin reduction assay; the reagent is an oxidized form of the redox indicator that is blue in color and nonfluorescent. When incubated with viable cells, the reagent is reduced and it changes its color from blue to red becoming fluorescent. Briefly, cells were seeded on gels with complete medium. After incubation times, the resazurin reagent was added directly to the culture medium with 10% volume of medium contained in each sample and incubated for 4 h at 37 °C with 5% CO_2_. Subsequently, aliquots from each sample were transferred to a 96 multiwell plate for fluorescence measurement at λ_exc_ 560 nm and λ_em_ 590 nm (Thermo Scientific Varioskan Flash Multimode Reader, Thermo Fisher Scientific, Waltham, Massachusetts, USA). We included a negative control of only medium without cells to determine the background signal and a positive control of 100% reduced resazurin reagent without cells.

## 4. Conclusions

We report here the preparation of strong supramolecular hydrogels that owe their peculiar properties to the introduction of graphene in the gelling mixture. These materials are strong, stable, thermoreversible, and elastic. The concentration of the gelator, the degree of graphene doping and the nature of the trigger are crucial to get hydrogels with the desired properties, where a high storage modulus coexists with good self-healing properties. Indeed, the hydrogels **1**–**8** (GdL as trigger, acidic pH) proved to be very strong but quite rigid, as testified by the analysis of the values of storage moduli and loss moduli variation during step strain experiments. In contrast, hydrogels **13**–**16** (gelator in 2% concentration, arginine as trigger, neutral pH) are very strong. SEM analysis of the corresponding aerogels demonstrates that the intimate structure of these hydrogels is formed by fibrous networks lacking in the other aerogels, which are characterized by locally oriented long strips that cross on the large scale. We also tested the biocompatibility of the most promising hydrogels **13**–**16** by assessing the bioavailability of mouse embryonic fibroblast (NIH-3T3) cells in the hydrogels, where pure water was replaced with complete medium serum. Cell adhesion on the hydrogel is strongly influenced by graphene doping. The hydrogel scaffold support cell growth at all the concentration of graphene doping.

In conclusion, in this paper, we demonstrated that the rheological properties and the biocompatibility of these graphene-hybrid hydrogels may be easily tuned selecting (i) the degree of the graphene doping and (ii) the nature of the gelator in the gelling mixture.

## Figures and Tables

**Table 1:** Hydrogels properties and numbering as a function of the gelator concentration (*w*/*w* concentration), graphene concentration (mg/mL), and trigger.

Entry	Trigger	Gelator A	Graphene (mg/mL)	pH	T_gel_^a^
**1**	GdL	1%—5 mg	0	4	70–77
**2**	GdL	1%—5 mg	0.5	4	79–81
**3**	GdL	1%—5 mg	1	4	77–79
**4**	GdL	1%—5 mg	5	4	80–89
**5**	GdL	2%—10 mg	0	4	98–100
**6**	GdL	2%—10 mg	0.5	4	94–99
**7**	GdL	2%—10 mg	1	3.5	98–100
**8**	GdL	2%—10 mg	5	3.5	80–87
**9**	Arg	1%—5 mg	0	7	73–79
**10**	Arg	1%—5 mg	0.5	7	78–96
**11**	Arg	1%—5 mg	1	7.5	85–91
**12**	Arg	1%—5 mg	5	7.5	91–100
**13**	Arg	2%—10 mg	0	7	90–100
**14**	Arg	2%—10 mg	0.5	8	86–100
**15**	Arg	2%—10 mg	1	8	92–100
**16**	Arg	2%—10 mg	5	8	95–100

^a^ The hydrogels are thermoreversible.

## Supplementary Materials

The following are available online. Figure S1: Photographs of hydrogels **1**–**16**, Figure S2: Frequency dependence of storage modulus (black) and loss modulus (red) for hydrogels **1**–**8**, Figure S3: Frequency dependence of storage modulus (black) and loss modulus (red) for hydrogels **9**–**16**, Figure S4: Values of storage moduli and loss moduli during a step strain experiment performed on hydrogels **1**–**8**, Figure S5: Values of storage moduli and loss moduli during a step strain experiment performed on hydrogels **9**–**16**, Figure S6: SEM images of aerogel obtained by freeze drying hydrogels samples **1**–**16**, Figure S7: Selected regions of ATR-IR spectra of aerogels **1**–**16**, Table S1: Summary of the Rheological properties of hydrogels **1**–**16**, Figure S8: Cell viability of NIH-3T3 in cellular medium after destruction of the hydrogel matrix.

## References

[1] Hoare T.R., Kohane D.S. (2008). Hydrogels in drug delivery: Progress and challenges. Polymer.

[2] Saul J.M., Williams D.F. (2013). Hydrogels in Regenerative Medicine. Handb. Polym. Appl. Med. Med. Devices.

[3] Van Vlierberghe S., Dubruel P., Schacht E. (2011). Biopolymer-based hydrogels as scaffolds for tissue engineering applications: A review. Biomacromolecules.

[4] Chakraborty P., Gazit E. (2018). Amino Acid Based Self-assembled Nanostructures: Complex Structures from Remarkably Simple Building Blocks. ChemNanoMat.

[5] Tibbitt M.W., Anseth K.S. (2009). Hydrogels as extracellular matrix mimics for 3D cell culture. Biotechnol. Bioeng..

[6] Seliktar D. (2012). Designing cell-compatible hydrogels for biomedical applications. Science.

[7] Wang N., Ma M., Luo Y., Liu T., Zhou P., Qi S., Xu Y., Chen H. (2018). Mesoporous Silica Nanoparticles-Reinforced Hydrogel Scaffold together with Pinacidil Loading to Improve Stem Cell Adhesion. ChemNanoMat.

[8] Barbalinardo M., Di Giosia M., Polishchuk I., Magnabosco G., Fermani S., Biscarini F., Calvaresi M., Zerbetto F., Pellegrini G., Falini G. (2019). Retinoic acid/calcite micro-carriers inserted in fibrin scaffolds modulate neuronal cell differentiation. J. Mater. Chem. B.

[9] Sangeetha N.M., Maitra U. (2005). Supramolecular gels: Functions and uses. Chem. Soc. Rev..

[10] Steed J.W. (2011). Supramolecular gel chemistry: Developments over the last decade. Chem. Commun..

[11] Appel E.A., Del Barrio J., Loh X.J., Scherman O.A. (2012). Supramolecular polymeric hydrogels. Chem. Soc. Rev..

[12] Jonker A.M., Löwik D.W.P.M., Van Hest J.C.M. (2012). Peptide- and protein-based hydrogels. Chem. Mater..

[13] Raeburn J., Cardoso A.Z., Adams D.J. (2013). The importance of the self-assembly process to control mechanical properties of low molecular weight hydrogels. Chem. Soc. Rev..

[14] Das D., Kar T., Das P.K. (2012). Gel-nanocomposites: Materials with promising applications. Soft Matter.

[15] Martí-Centelles R., Escuder B. (2018). Morphology Diversity of L-Phenylalanine-Based Short Peptide Supramolecular Aggregates and Hydrogels. ChemNanoMat.

[16] Das T., Häring M., Haldar D., Díaz Díaz D. (2018). Phenylalanine and derivatives as versatile low-molecular-weight gelators: Design, structure and tailored function. Biomater. Sci..

[17] Memic A., Alhadrami H.A., Hussain M.A., Aldhahri M., Al Nowaiser F., Al-Hazmi F., Oklu R., Khademhosseini A. (2015). Hydrogels 2.0: Improved properties with nanomaterial composites for biomedical applications. Biomed. Mater..

[18] Gaharwar A.K., Peppas N.A., Khademhosseini A. (2014). Nanocomposite hydrogels for biomedical applications. Biotechnol. Bioeng..

[19] Iglesias D., Bosi S., Melchionna M., Da Ros T., Marchesan S. (2016). The Glitter of Carbon Nanostructures in Hybrid/Composite Hydrogels for Medicinal Use. Curr. Top. Med. Chem..

[20] Shin S.R., Bae H., Cha J.M., Mun J.Y., Chen Y.-C., Tekin H., Shin H., Farshchi S., Dokmeci M.R., Tang S. (2012). Carbon nanotube reinforced hybrid microgels as scaffold materials for cell encapsulation. ACS Nano.

[21] Komatsu H., Ikeda M., Hamachi I. (2011). Mechanical Reinforcement of Supramolecular Hydrogel through Incorporation of Multiple Noncovalent Interactions. Chem. Lett..

[22] Sugikawa K., Inoue Y., Kozawa K., Ikeda A. (2018). Introduction of Fullerenes into Hydrogels via Formation of Fullerene Nanoparticles. ChemNanoMat.

[23] Paul S., Basu K., Das K.S., Banerjee A. (2018). Peptide-Based Hydrogels as a Scaffold for In Situ Synthesis of Metal Nanoparticles: Catalytic Activity of the Nanohybrid System. ChemNanoMat.

[24] Shin S.R., Jung S.M., Zalabany M., Kim K., Zorlutuna P., Kim S.B., Nikkhah M., Khabiry M., Azize M., Kong J. (2013). Carbon-nanotube-embedded hydrogel sheets for engineering cardiac constructs and bioactuators. ACS Nano.

[25] Iglesias D., Melle-Franco M., Kurbasic M., Melchionna M., Abrami M., Grassi M., Prato M., Marchesan S. (2018). Oxidized Nanocarbons-Tripeptide Supramolecular Hydrogels: Shape Matters!. ACS Nano.

[26] Wang H., Chen Q., Zhou S. (2018). Carbon-based hybrid nanogels: A synergistic nanoplatform for combined biosensing, bioimaging, and responsive drug delivery. Chem. Soc. Rev..

[27] Guidetti G., Giuri D., Zanna N., Calvaresi M., Montalti M., Tomasini C. (2018). Biocompatible and Light-Penetrating Hydrogels for Water Decontamination. ACS Omega.

[28] Adhikari B., Banerjee A. (2011). Short peptide based hydrogels: Incorporation of graphene into the hydrogel. Soft Matter.

[29] González-Domínguez J.M., Martín C., Durá Ó.J., Merino S., Vázquez E. (2018). Smart Hybrid Graphene Hydrogels: A Study of the Different Responses to Mechanical Stretching Stimulus. ACS Appl. Mater. Interfaces.

[30] Wychowaniec J.K., Iliut M., Zhou M., Moffat J., Elsawy M.A., Pinheiro W.A., Hoyland J.A., Miller A.F., Vijayaraghavan A., Saiani A. (2018). Designing peptide/graphene hybrid hydrogels through fine tuning of molecular interactions. Biomacromolecules.

[31] Das Mahapatra R., Dey J., Weiss R.G. (2019). Poly(vinyl alcohol)-induced thixotropy of an L-carnosine-based cytocompatible, tripeptidic hydrogel. Soft Matter.

[32] Kenry L.W., Loh K.P., Lim C.T. (2018). When stem cells meet graphene: Opportunities and challenges in regenerative medicine. Biomaterials.

[33] Yang Y., Asiri A.M., Tang Z., Du D., Lin Y. (2013). Graphene based materials for biomedical applications. Mater. Today.

[34] Kumar S., Chatterjee K. (2016). Comprehensive Review on the Use of Graphene-Based Substrates for Regenerative Medicine and Biomedical Devices. ACS Appl. Mater. Interfaces.

[35] Chung C., Kim Y.K., Shin D., Ryoo S.R., Hong B.H., Min D.H. (2013). Biomedical applications of graphene and graphene oxide. Acc. Chem. Res..

[36] Wu J., Chen A., Qin M., Huang R., Zhang G., Xue B., Wei J., Li Y., Cao Y., Wang W. (2015). Hierarchical construction of a mechanically stable peptide-graphene oxide hybrid hydrogel for drug delivery and pulsatile triggered release in vivo. Nanoscale.

[37] Bai H., Li C., Wang X., Shi G. (2010). A pH-sensitive graphene oxide composite hydrogel. Chem. Commun..

[38] Zu S.-Z., Han B.-H. (2009). Aqueous Dispersion of Graphene Sheets Stabilized by Pluronic Copolymers: Formation of Supramolecular Hydrogel. J. Phys. Chem. C.

[39] Gao H., Sun Y., Zhou J., Xu R., Duan H. (2013). Mussel-inspired synthesis of polydopamine-functionalized graphene hydrogel as reusable adsorbents for water purification. ACS Appl. Mater. Interfaces.

[40] Cong H.P., Wang P., Yu S.H. (2013). Stretchable and self-healing graphene oxide-polymer composite hydrogels: A dual-network design. Chem. Mater..

[41] Guo H., Jiao T., Zhang Q., Guo W., Peng Q., Yan X. (2015). Preparation of Graphene Oxide-Based Hydrogels as Efficient Dye Adsorbents for Wastewater Treatment. Nanoscale Res. Lett..

[42] Calvaresi M., Zerbetto F. (2013). The devil and holy water: Protein and carbon nanotube hybrids. Acc. Chem. Res..

[43] Marchesan S., Prato M. (2015). Under the lens: Carbon nanotube and protein interaction at the nanoscale. Chem. Commun..

[44] Sloan A.W.N., Santana-Pereira A.L.R., Goswami J., Liles M.R., Davis V.A. (2017). Single-Walled Carbon Nanotube Dispersion in Tryptic Soy Broth. ACS Macro Lett..

[45] Di Giosia M., Valle F., Cantelli A., Bottoni A., Zerbetto F., Fasoli E., Calvaresi M. (2019). High-throughput virtual screening to rationally design protein—Carbon nanotube interactions. Identification and preparation of stable water dispersions of protein—Carbon nanotube hybrids and efficient design of new functional materials. Carbon N. Y..

[46] Angulo-Pachón C.A., Díaz-Oltra S., Ojeda-Flores J.J., Falomir E., Galindo F., Miravet J.F. (2018). Self-Assembled Nanofibrilar Networks: Boosting Hydrogelation Efficiency by Replacement of a Pyridine Moiety by a Quinoline One. ChemNanoMat.

[47] Zanna N., Merlettini A., Tatulli G., Milli L., Focarete M.L., Tomasini C. (2015). Hydrogelation Induced by Fmoc-Protected Peptidomimetics. Langmuir.

[48] Milli L., Zanna N., Merlettini A., Di Giosia M., Calvaresi M., Focarete M.L., Tomasini C. (2016). Pseudopeptide-Based Hydrogels Trapping Methylene Blue and Eosin Y. Chem. Eur. J..

[49] Zanna N., Merlettini A., Tomasini C. (2016). Self-healing hydrogels triggered by amino acids. Org. Chem. Front..

[50] Tomasini C., Villa M. (2001). Pyroglutamic acid as a pseudoproline moiety: A facile method for its introduction into polypeptide chains. Tetrahedron Lett..

[51] Lucarini S., Tomasini C. (2001). Synthesis of oligomers of trans-(4S,5R)-4-carboxybenzyl 5-methyl oxazolidin-2-one: An approach to new foldamers. J. Org. Chem..

[52] Angelici G., Falini G., Hofmann H.-J., Huster D., Monari M., Tomasini C. (2008). A Fiberlike Peptide Material Stabilized by Single Intermolecular Hydrogen Bonds. Angew. Chem. Int. Ed..

[53] Angelici G., Falini G., Hofmann H.J., Huster D., Monari M., Tomasini C. (2009). Nanofibers from oxazolidi-2-one containing hybrid foldamers: What is the right molecular size?. Chem. Eur. J..

[54] Zanna N., Focaroli S., Merlettini A., Gentilucci L., Teti G., Falconi M., Tomasini C. (2017). Thixotropic Peptide-Based Physical Hydrogels Applied to Three-Dimensional Cell Culture. ACS Omega.

[55] Tomasini C., Zanna N. (2017). Oxazolidinone-containing pseudopeptides: Supramolecular materials, fibers, crystals, and gels. Biopolymers.

[56] Castellucci N., Falini G., Angelici G., Tomasini C. (2011). Formation of gels in the presence of metal ions. Amino Acids.

[57] Milli L., Castellucci N., Tomasini C. (2014). Turning Around the L-Phe-D-Oxd Moiety for a Versatile Low-Molecular-Weight Gelator. Eur. J. Org. Chem..

[58] Otsuka T., Maeda T., Hotta A. (2014). Effects of salt concentrations of the aqueous peptide-amphiphile solutions on the sol-gel transitions, the gelation speed, and the gel characteristics. J. Phys. Chem. B.

[59] Chen L., McDonald T.O., Adams D.J. (2013). Salt-induced hydrogels from functionalised-dipeptides. RSC Adv..

[60] Adams D.J., Butler M.F., Frith W.J., Kirkland M., Mullen L., Sanderson P. (2009). A new method for maintaining homogeneity during liquid–hydrogel transitions using low molecular weight hydrogelators. Soft Matter.

[61] Adams D.J., Mullen L.M., Berta M., Chen L., Frith W.J. (2010). Relationship between molecular structure, gelation behaviour and gel properties of Fmoc-dipeptides. Soft Matter.

[62] Fichman G., Guterman T., Adler-abramovich L., Gazit E. (2015). Synergetic functional properties of two-component single amino acid-based hydrogels. CrystEngComm.

[63] Yang Z., Liang G., Xu B. (2008). Enzymatic hydrogelation of small molecules. Acc. Chem. Res..

[64] Liyanage W., Vats K., Rajbhandary A., Benoit D.S.W., Nilsson B.L. (2015). Multicomponent dipeptide hydrogels as extracellular matrix-mimetic scaffolds for cell culture applications. Chem. Commun..

[65] Pramanik A., Paikar A., Haldar D. (2015). Sonication-induced instant fibrillation and fluorescent labeling of tripeptide fibers. RSC Adv..

[66] Castellucci N., Angelici G., Falini G., Monari M., Tomasini C. (2011). L-Phe-D-Oxd: A privileged scaffold for the formation of supramolecular materials. European J. Org. Chem..

[67] Draper E.R., Adams D.J. (2017). Low-Molecular-Weight Gels: The State of the Art. Chem.

[68] Colquhoun C., Draper E., Schweins R., Marcello M., Serpell L., Vadukul D., Adams D. (2017). Controlling the Network Type in Self-Assembled Dipeptide Hydrogels. Soft Matter.

[69] Basak S., Nanda J., Banerjee A. (2014). Multi-stimuli responsive self-healing metallo-hydrogels: Tuning of the gel recovery property. Chem. Commun..

[70] Karan C.K., Bhattacharjee M. (2016). Self-Healing and Moldable Metallogels as the Recyclable Materials for Selective Dye Adsorption and Separation. ACS Appl. Mater. Interfaces.

[71] Roy S., Baral A., Banerjee A. (2013). An amino-acid-based self-healing hydrogel: Modulation of the self-healing properties by incorporating carbon-based nanomaterials. Chem. Eur. J..

[72] Liu Y., Ling S., Wang S., Chen X., Shao Z. (2014). Thixotropic silk nanofibril-based hydrogel with extracellular matrix-like structure. Biomater. Sci..

[73] Foster J.S., Prentice A.W., Forgan R.S., Paterson M.J., Lloyd G.O. (2018). Targetable Mechanical Properties by Switching between Self-Sorting and Co-assembly with In Situ Formed Tripodal Ketoenamine Supramolecular Hydrogels. ChemNanoMat.

[74] Gong Z., Yang Y., Ren Q., Chen X., Shao Z. (2012). Injectable thixotropic hydrogel comprising regenerated silk fibroin and hydroxypropylcellulose. Soft Matter.

[75] Pek Y.S., Wan A.C.A., Ying J.Y. (2010). The effect of matrix stiffness on mesenchymal stem cell differentiation in a 3D thixotropic gel. Biomaterials.

[76] Li Y., Zhou F., Wen Y., Liu K., Chen L., Mao Y., Yang S., Yi T. (2014). (−)-Menthol based thixotropic hydrogel and its application as a universal antibacterial carrier. Soft Matter.

[77] Beckert F., Trenkle S., Thomann R., Mülhaupt R. (2014). Mechanochemical Route to Functionalized Graphene and Carbon Nanofillers for Graphene/SBR Nanocomposites. Macromol. Mater. Eng..

