# Supplementary material for: Tuning Mechanical Properties of Pseudopeptide Supramolecular Hydrogels by Graphene Doping
Source: Molecules. 2019 Nov 28;24(23):4345. doi: 10.3390/molecules24234345 (PMC6930602; doi:10.3390/molecules24234345)
Supplement: Supplementary file 1 [file molecules-24-04345-s001.pdf]

Supporting Information

Tuning Pseudopeptide Supramolecular Hydrogels Mechanical Properties by Graphene Doping

Demetra Giuri [1], Marianna Barbalinardo [2], Nicola Zanna [1], Paolo Paci [1], Marco Montalti [1], Massimiliano Cavallini [2], Francesco Valle [2], Matteo Calvaresi [1]*, Claudia Tomasini [1] *

[1] Dipartimento di Chimica "Giacomo Ciamician" Università di Bologna - Via Selmi, 2 - 40126 Bologna - Italy; claudia.tomasini@unibo.it; matteo.calvaresi3@unibo.it

[2] Istituto per lo Studio dei Materiali Nanostrutturati, Consiglio Nazionale delle Ricerche, (ISMN-CNR) - Via P. Gobetti 101 – 40129 Bologna – Italy

Contents

**Figure S1.** Photographs of hydrogels **1-16**	Page S1
**Table S1**. Summary of the Rheological properties of hydrogels **1-16**	Page S2
**Figure S2.** Frequency dependence of storage modulus (black) and loss modulus (red) for hydrogels **1-8**	Page S3
**Figure S3.** Frequency dependence of storage modulus (black) and loss modulus (red) for hydrogels **9-16**	Page S4
**Figure S4.** Values of storage moduli and loss moduli during a step strain experiment performed on hydrogels **1-8**	Page S5
**Figure S5.** Values of storage moduli and loss moduli during a step strain experiment performed on hydrogels **9-16**	Page S6
**Figure S6.** SEM images of aerogel obtained by freeze drying hydrogels samples **1-16**	Page S7
**Figure S7.** Selected regions of ATR-IR spectra of aerogels **1-16**	Page S8
**Figure S8.** Cell viability of NIH-3T3 in cellular medium after destruction of the hydrogel matrix.	Page S9

**Trigger**	**% Graphene**			
	**0**	**0.5**	**1**	**5**
**GdL (1% gelator Conc.)**	**1** 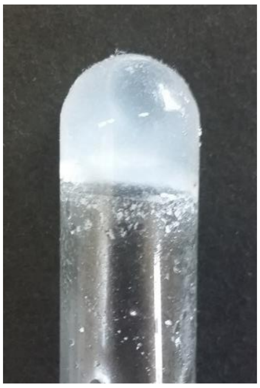	**2** 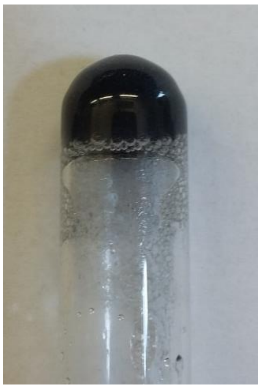	**3** 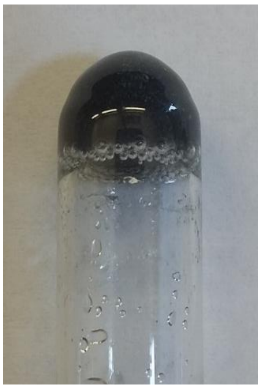	**4** 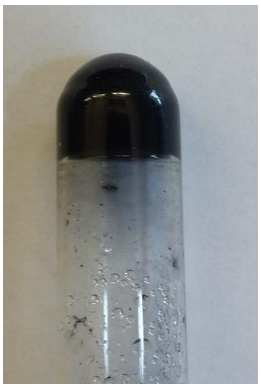
**GdL (2% gelator Conc.)**	**5** 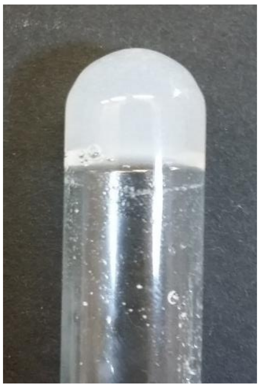	**6** 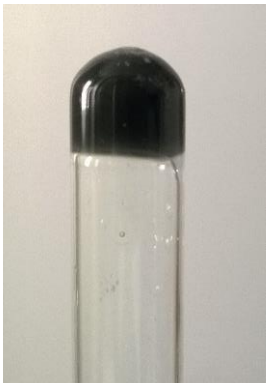	**7** 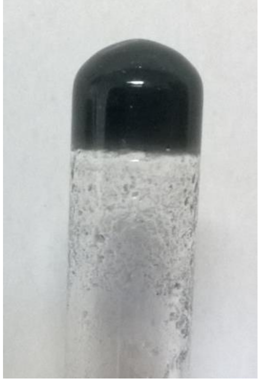	**8** 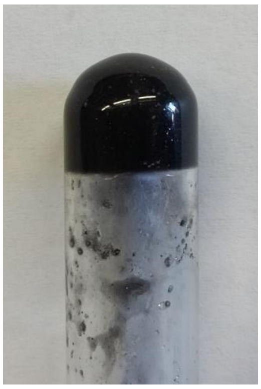
**Arg (1% gelator Conc.)**	**9** 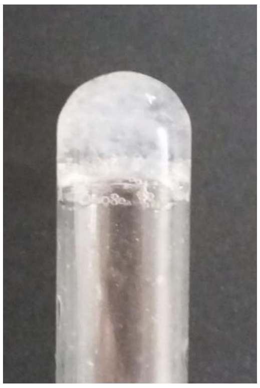	**10** 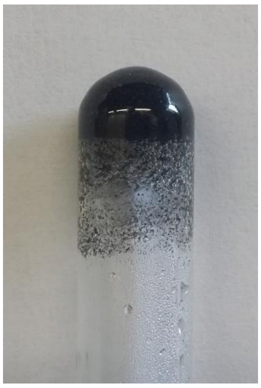	**11** 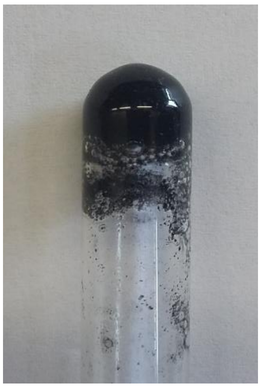	**12** 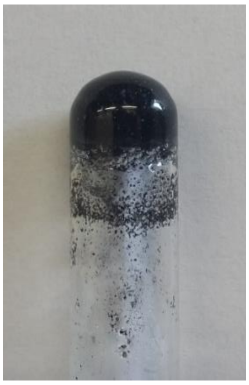
**Arg (2% gelator Conc.)**	**13** 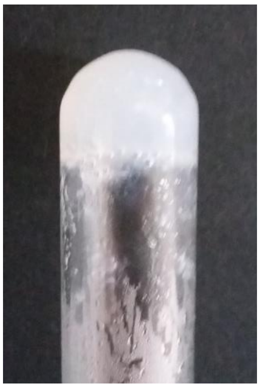	**14** 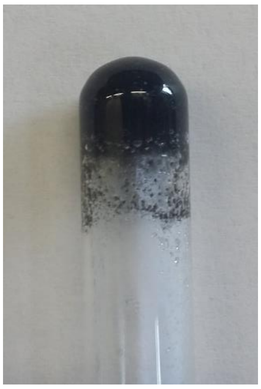	**15** 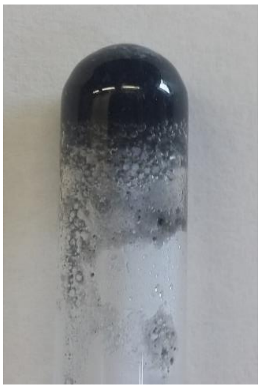	**16** 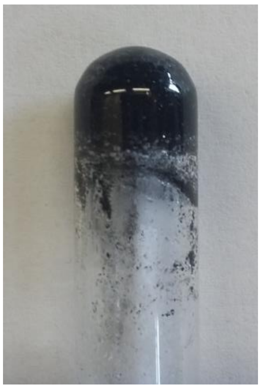

Figure S1. Photographs of hydrogels **1**-**16.**

Table S1. Summary of the Rheological properties of hydrogels **1**-**16**.

Entry	Trigger	G’ (Pa)	G’’ (Pa)
**1**	GdL	34300-35200	1100-2900
**2**	GdL	30000-33300	1000-4300
**3**	GdL	45700-48300	1500-4100
**4**	GdL	75000-84200	3700-5900
**5**	GdL	31000-36000	3200-4200
**6**	GdL	46700-47700	2600-4300
**7**	GdL	50600-54900	2500-5100
**8**	GdL	64000-75900	4200-7600
**9**	Arg	20900-26400	3100-3500
**10**	Arg	39600-42300	900-4700
**11**	Arg	42700-49300	2400-3200
**12**	Arg	75800-77700	5800-7900
**13**	Arg	62000-72600	4400-12000
**14**	Arg	78200-86300	4200-6900
**15**	Arg	110400-110700	6400-13100
**16**	Arg	112000-129000	7100-12200

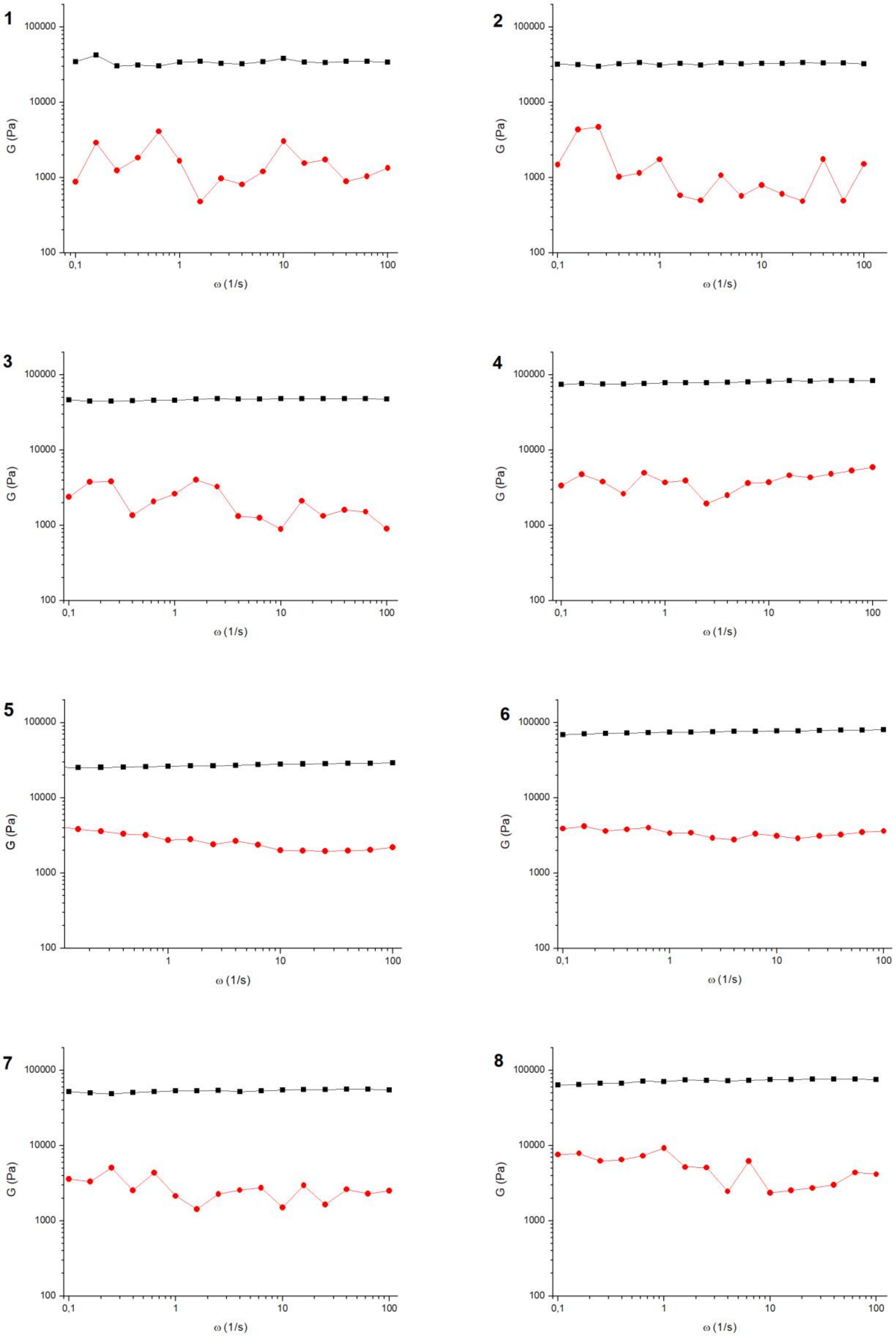

Figure S2. Frequency dependence of storage modulus (black) and loss modulus (red) for hydrogels **1-8**.

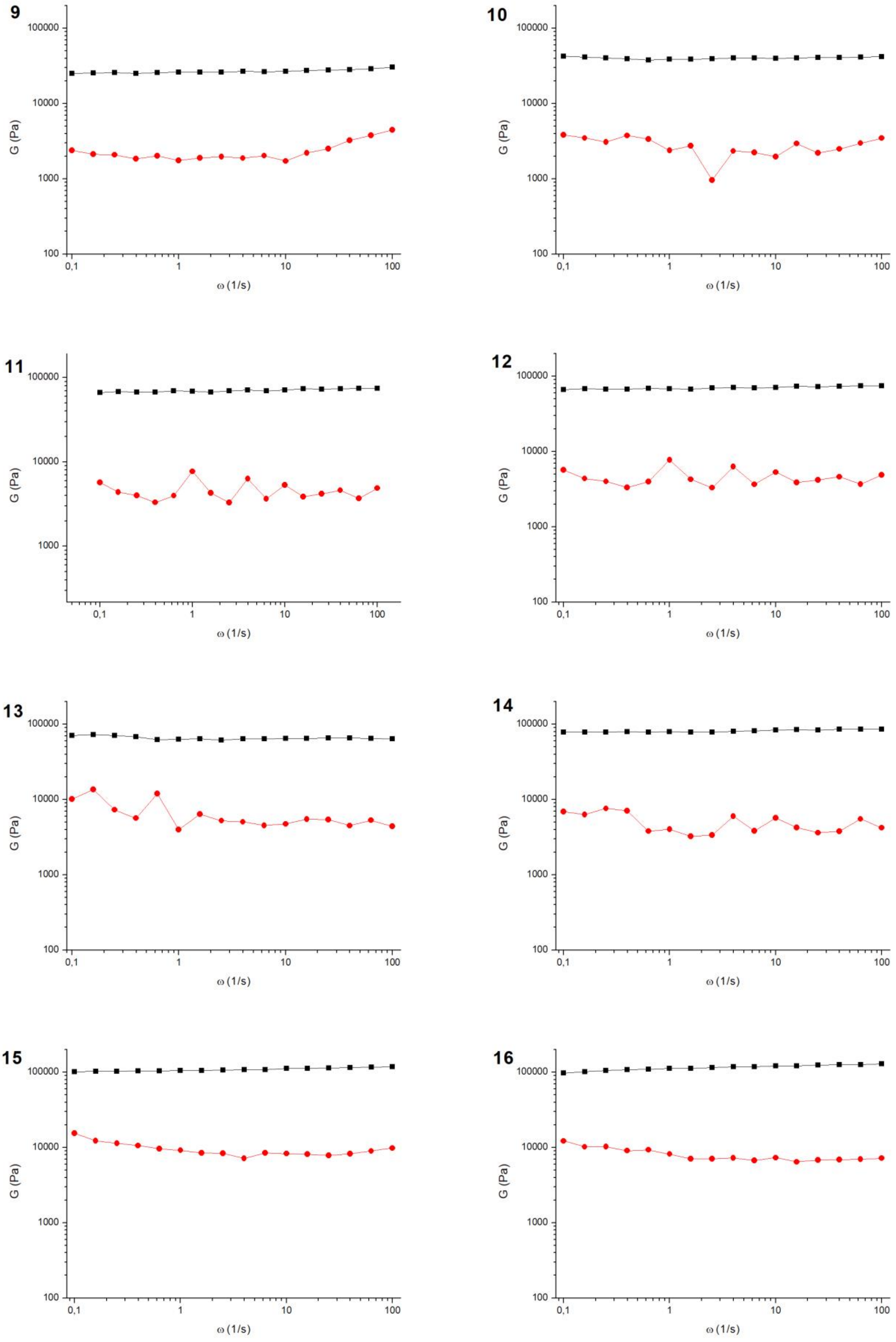

Figure S3. Frequency dependence of storage modulus (black) and loss modulus (red) for hydrogels **9-16**.

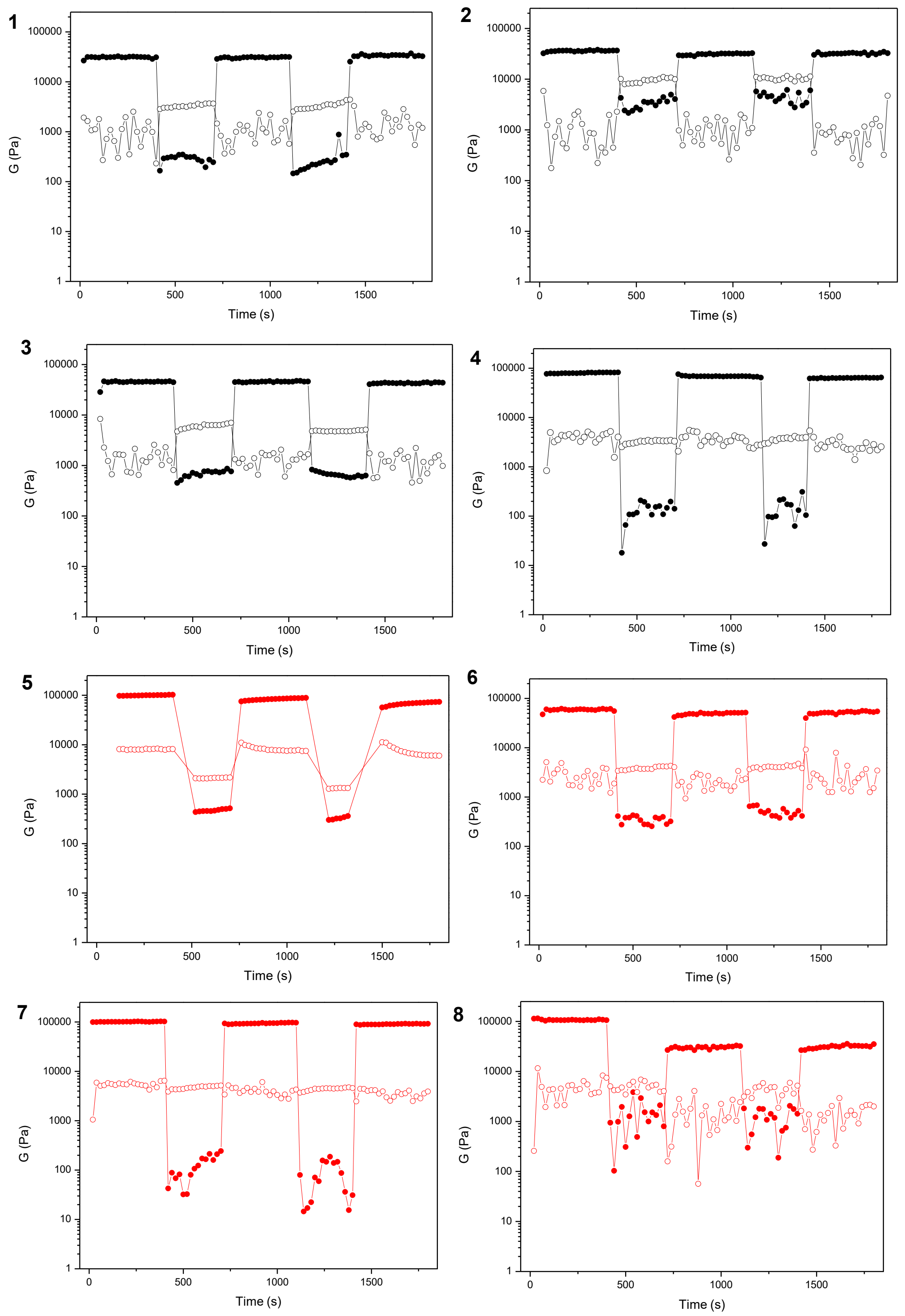

Figure S4. Values of storage moduli (solid circles) and loss moduli (empty circles) during a step strain experiment performed on hydrogels **1-8**.

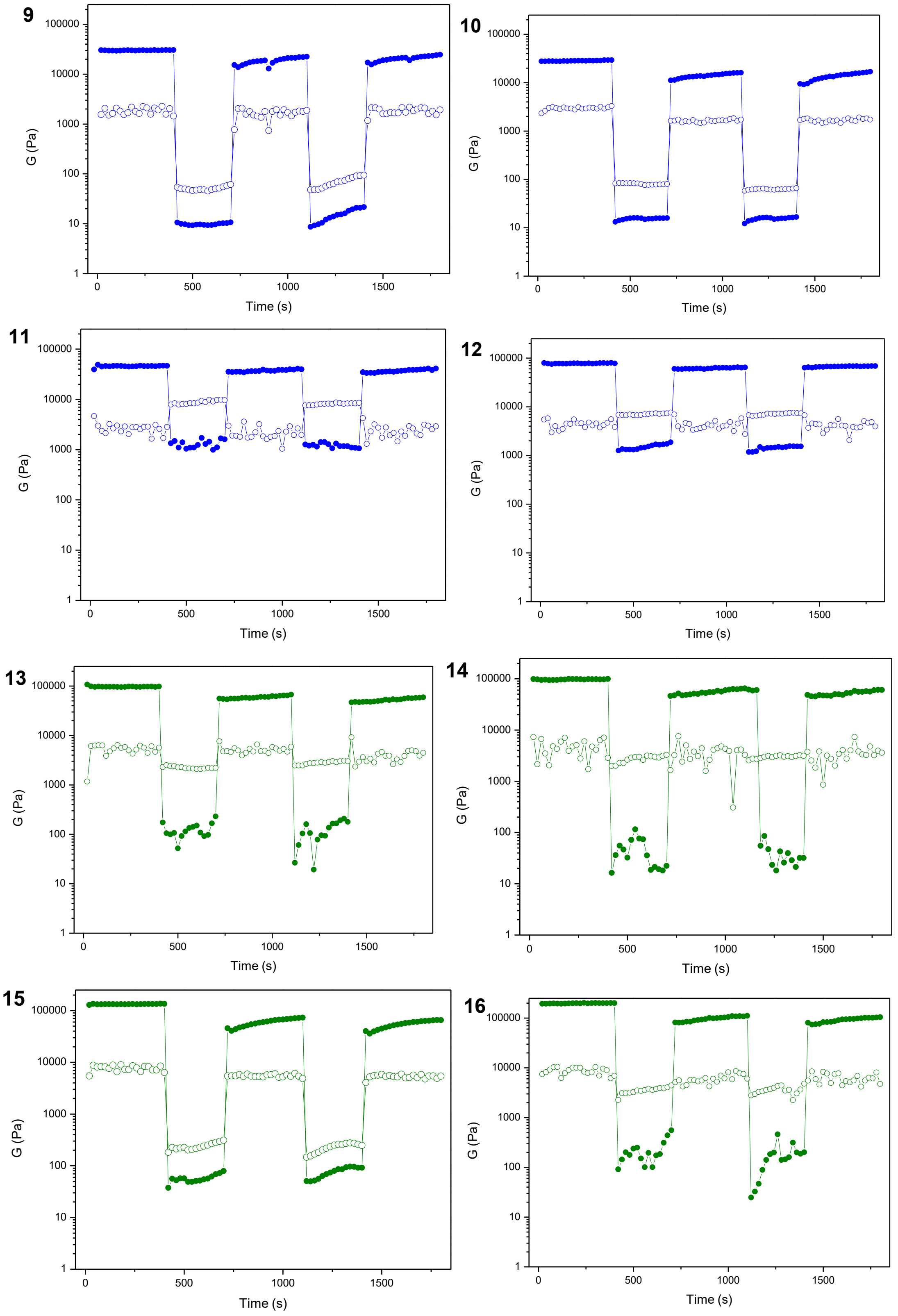

Figure S5. Values of storage moduli (solid circles) and loss moduli (empty circles) during a step strain experiment performed on hydrogels **9-16**.

Trigger	% Graphene			
	0	0.5	1	5
GdL (1% gelator Conc.)	**1** 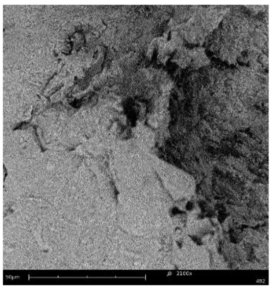	**2** 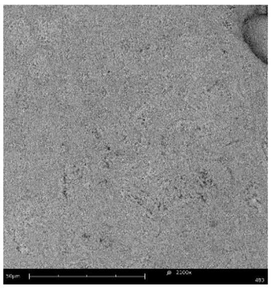	**3** 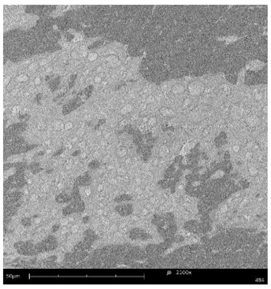	**4** 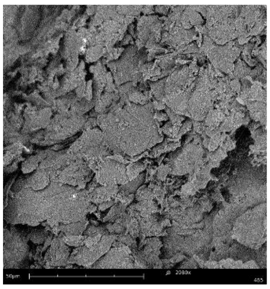
GdL (2% gelator Conc.)	**5** 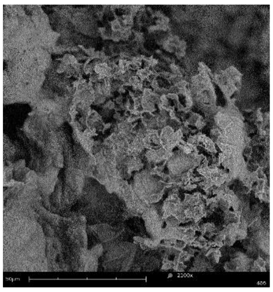	**6** 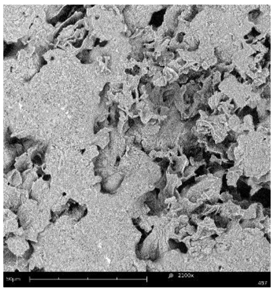	**7** 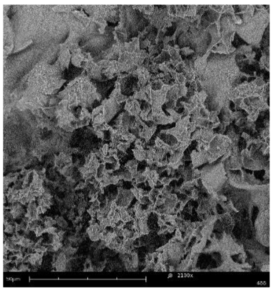	**8** 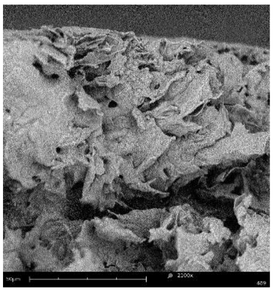
Arg (1% gelator Conc.)	**9** 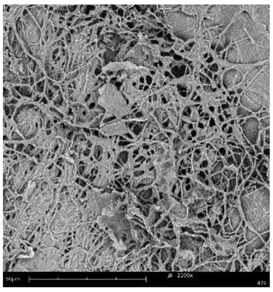	**10** 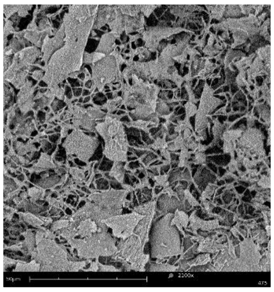	**11** 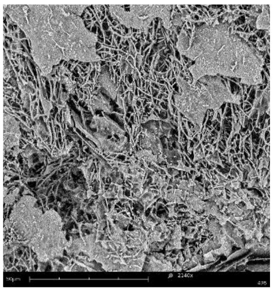	**12** 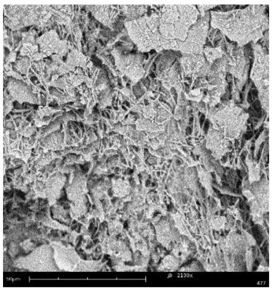
Arg (2% gelator Conc.)	**13** 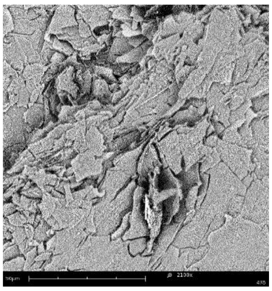	**14** 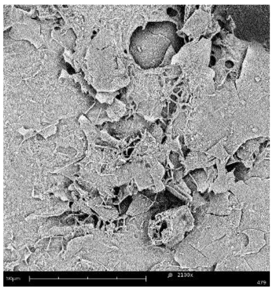	**15** 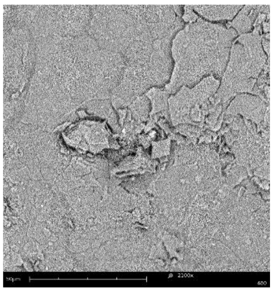	**16** 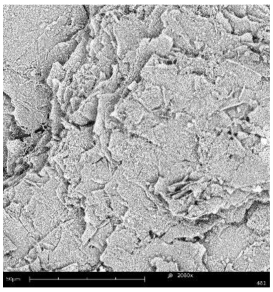

Figure S6. SEM images of aerogel obtained by freeze drying hydrogels samples **1**-**16**.

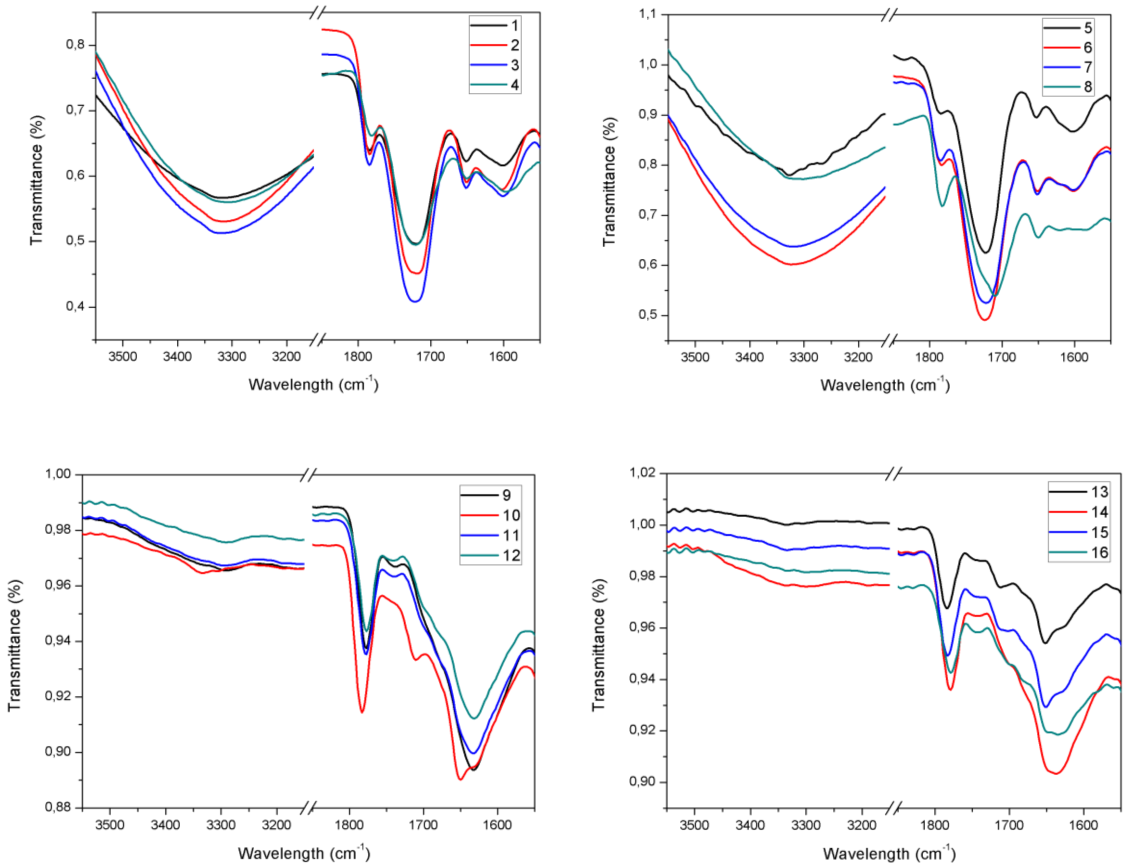

Figure S7. Selected regions of ATR-IR spectra of aerogels **1**-**16**.

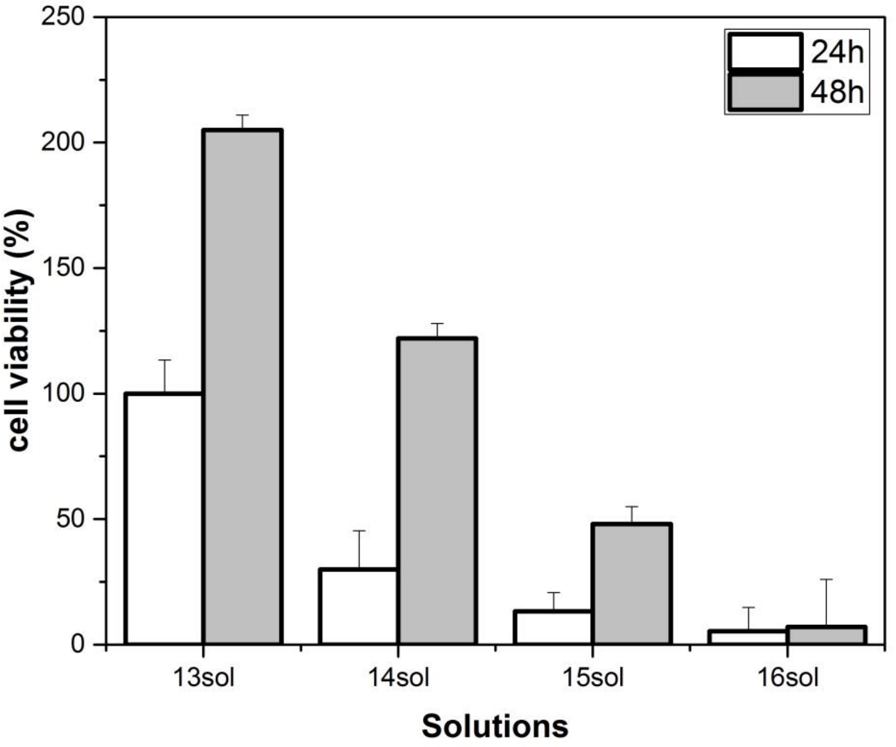

Figure S8. Cell viability of NIH-3T3 in cellular medium solution. We tested (from left to right): **13:** cellular medium added with gelator (2%); **14:** cellular medium added with graphene (0.5 mg), **15:** cellular medium added with graphene (1 mg), **16:** cellular medium added with graphene (5 mg). Data represent the mean ± standard deviation.
